# Malaria transmission pattern across the Sahelian, humid savanna, highland and forest eco-epidemiological settings in Cameroon

**DOI:** 10.1186/s12936-023-04544-z

**Published:** 2023-04-07

**Authors:** Nelly Armanda Kala Chouakeu, Timoléon Tchuinkam, Roland Bamou, Mabu Maxim Bindamu, Abdou Talipouo, Edmond Kopya, Parfait Awono-Ambene, Christophe Antonio-Nkondjio

**Affiliations:** 1 Vector Borne Diseases Laboratory of the Research Unit of Biology and Applied Ecology (VBID-RUBAE), Department of Animal Biology, Faculty of Science of the University of Dschang, Dschang, Cameroon; 2 Organisation de Coordination Pour la Lutte Contre les Endémies en Afrique Centrale (OCEAC), Yaoundé, Cameroon; 3 Laboratory of Parasitology and Ecology, Faculty of Sciences, University of Yaoundé, Yaoundé, Cameroon; 4 University of Bamenda, Bamenda, Cameroon

**Keywords:** Malaria, Transmission, Anopheles, Eco-epidemiological settings, Cameroon

## Abstract

**Background:**

Malaria remains a major public health concern in Cameroon. Understanding vector distribution and malaria transmission dynamics is of paramount importance for evaluating the performance of control strategies. This study assesses patterns of malaria transmission in four eco-epidemiological settings in Cameroon.

**Methods:**

Adult mosquitoes were collected using Human Landing Catches (HLC) once every 4 months from August 2019 to November 2021 in Kaélé, Tibati, Santchou and Bertoua. Mosquitoes were sorted by genus and *Anopheles gambiae *sensu lato (*s.l*.) species complex were identified using PCR. The presence of *Plasmodium falciparum* circumsporozoite protein (CSP) was measured by ELISA; the entomological inoculation rates (EIR) was estimated in each locality.

**Results:**

A total of 23,536 mosquitoes were collected. *Anopheles* *gambiae* and/or *Anopheles* *coluzzii* were the main malaria vectors in all sites. *Anopheles* *arabiensis* was recorded in low frequency in Kaélé and Tibati. Other species collected included *Anopheles funestus, Anopheles* *pharoensis* and *Anopheles ziemmani*. High anopheline biting rates were recorded outdoor in all sites except in Kaélé. Important differences in species biting dynamics were observed between sites. The sporozoite infection rate varied from 0.36 to 4%. The daily EIR was found to vary from 0.07 in Santchou to 0.26 infected bites/man/night (ib/m/n) in Kaélé).

**Conclusion:**

The study suggests heterogeneous patterns of malaria transmission in different ecoepidemiological settings across the country. The findings stress the need to improve malaria vector control strategies.

**Supplementary Information:**

The online version contains supplementary material available at 10.1186/s12936-023-04544-z.

## Background

Despite more than a century of malaria research, the disease remains a major public health concern in Africa [1]. It is estimated that about 241 million people across the world suffer from malaria yearly, with 627,000 associated deaths registered in 2021 [1]. The implementation of key malaria prevention measures such as the massive scale up of ITNs has played significant role in decreasing the burden of malaria between 2010 and 2015 [2]. Since 2017, the downward trend in morbidity and mortality has slowed and even reversed with the COVID-19 pandemic which has limited the delivery of essential commodities used for malaria prevention or case management [1].

In Cameroon, malaria represents a major public health concern with the entire country being at risk of transmission [3]. In 2019, up to 2638191 cases were recorded in health care facilities with 4500 associated deaths. Children under 5 years are the most affected with 65% of deaths among the total number of death caused by malaria [3]. The national vector control policy is based primarily on the widespread use of insecticide-treated nets (ITNS) [3]. It is estimated that, more than 80% of the population possess a treated bed net [4–7]. Unfortunately, several factors still affect the performance of ITNs, such as changes in vector feeding and resting behaviour and the rapid spread of insecticide resistance in malaria vector populations [8–12]. The success of vector control strategies requires up to date information on vector population distribution, bionomic and malaria transmission pattern [9–11].

A high number of species contribute to malaria transmission across the country [8, 13–16]. Variations in the distribution and behavior of these vector species affect vector control [11, 13, 14]. Species such as *Anopheles gambiae *sensu lato (*s.l*.) despite displaying high endophilic and endophagic behavior can become exophilic or exophagic in response to the use of impregnated mosquito nets [17]. It is now clear that the use of indoor based interventions alone will not achieve malaria elimination if outdoor biting or resting vectors are not targeted [18].

Although, increasing efforts have been made to characterize malaria transmission patterns across several epidemiological settings in Cameroon [6, 11, 13–15, 19–29], much is still left to be done. There is paucity of data in some region including the deep Eastern forest region, the Adamawa and the western highlands region. This information could be crucial for improving malaria control strategies and filling important knowledge gaps which are impeding control efforts. This study presents data on vector distribution and malaria transmission pattern in four eco-epidemiological settings in Cameroon: the Sahelian zone (Kaélé), the humid savannah area (Tibati), the West highland area covered by grassfields (Santchou), and the forest zone (Bertoua).

## Methods

### Study sites

**Fig. 1:**
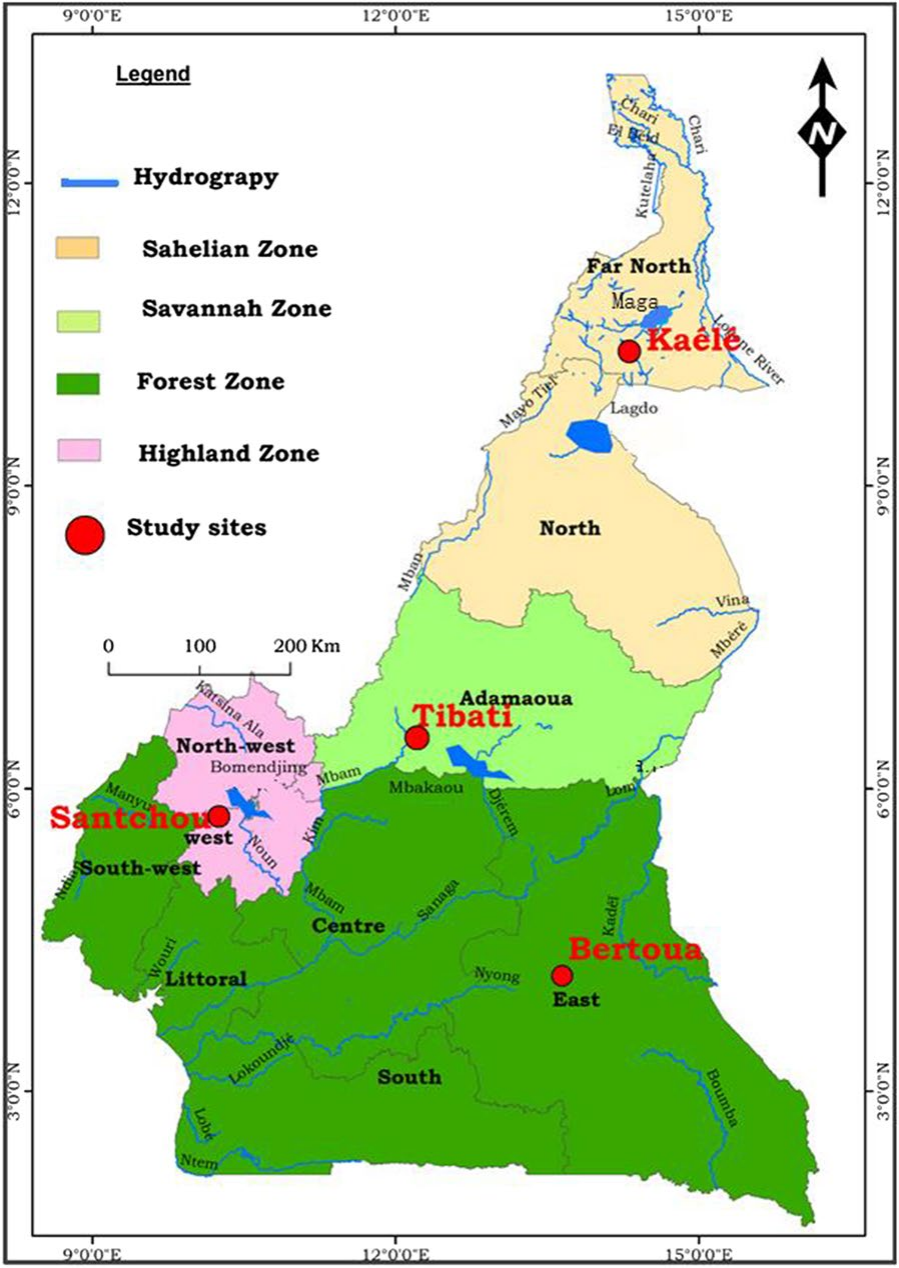
Map of the study sites

The study was conducted from July 2019 to September 2021 in four epidemiological settings in Cameroon: Kaélé in the Sahelian zone, Tibati in the Sahelo-soudanese area, Bertoua in the forest area and Santchou in the western highlands. The characteristics of the different study sites are presented in Table 1 and Fig. 1.

**Table 1:** Description of the study sites

Characteristics	Kaélé	Tibati	Bertoua	Santchou
Region	Far North	Adamawa	East	West
Geographic coordinates	10° 50′ 36’’ N; 14° 56′ 23’’ E	6° 28′ 00’’N; 12° 38′ 00’’ E	4° 34′ 30’’N; 13° 41′ 04’’E	5° 16′ 55’’ N; 9° 58′ 27’’E
Ecological domain	Sahelian	Sahelo-Sudanese (humid savannah)	Forest	Highland Grassfields
Urban/rural	Rural	Rural	Urban	Rural
Altitude (above sea level)	304 m	840 m	400 m	1000 m
Climate	Sahelian	Tropical humid	Subtropical	Equatorial
Average temperature (°C)	33	28	26	23
Average rainfall(mm)	726.2	951.9	1750	1364.4
Seasons	9 months dry season /3 months rainy season	6 months dry/6 months rainy season	Two rainy seasons (8 months); two dry (4 months)	Two rainy seasons (8 months) two dry seasons (4 months)
Vegetation	Wooded Savanah	Grassy Savanah	dense forest	Grassfields

Kaélé is a locality of over 127000, close to the Maga dam on the Logone river. Malaria situation in the area could possibly be affected by the frequent movements of the population between Cameroon and Chad. The region belongs to the Sahelian domain characterize by a long dry season running from October to May with only 4 months of rains, going from June to September.

Tibati is a locality of over 36,000 inhabitants, situated midway between the north and the south of the country close to the Mbakaou dam on the Sanaga river. The region belongs to the Sahelo-Sudanese domain characterize by two seasons, a dry season extending from November to February and a rainy season from March to October. Although considered as highly endemic for malaria, there is little data on malaria transmission pattern and vector distribution.

Bertoua is a city of over 200 000 inhabitants situated in the equatorial forest zone with several river systems: the Nyong, the Dja, the Lom, the Kadeï, the Boumba and Ngoko. The region belongs to the Sahelo-Sudanese domain characterized by four seasons including two rainy seasons and two dry season. A long rainy season from September to November, a long dry season from November to March, a short rainy season from March to June and a short dry season from July to August. Previous studies indicated a high frequency of insecticide resistance in vector populations [30, 31]. The region is also one of the most affected by malaria in the country [3].

Santchou is a locality of over 41,000 inhabitants, situated in the highland zone. The region is characterize by four seasons including a long rainy season from mid- August to October; a short rainy season from March to June; a long dry season from mid-October to March and a short dry season from June to Mid-August. Santchou is an area with intensive practice of agriculture and frequent use of pesticides [24, 32]. The river Nkam and its tributaries provide a dense hydrography in the locality.

### Mosquito collections and processing

Adult mosquitoes were sampled from August 2019 to November 2021 using Human Landing Catches (HLC). A total of six surveys were conducted in Santchou and Bertoua (August 2019, November 2019, July 2020, November 2020, May 2021, August 2021) and five in Tibati and Kaélé (August 2019, November 2019, November 2020, May 2021, August 2021). In each site, mosquito collection was carried out both indoors and outdoors in four randomly selected houses between 07:00 pm and 06:00 am. Mosquito collection was undertaken during two consecutive nights. Each mosquito collector worked half a night, from 7 pm to 1 am or from 1 to 6 am. A circular permutation of the place of collection and of the time slots was established for each of the collector in order to reduce the effects of individual factors. After mosquito collection, culicines were identified and separated from anophelines. Anopheline species were identified using keys for the morphological identification of Gillies and Coetzee [33]. *Anopheles* specimens were stored individually in labelled Eppendorf tubes containing a desiccant, then transported to the Malaria Research Laboratory of the OCEAC for further analysis.

### Laboratory processing of mosquitoes

#### Detection of *Plasmodium* parasite infections in mosquitoes

The heads and thoraces of female anophelines were tested for the presence of circumsporozoite protein using enzyme-linked immunosorbent assay (ELISA) according to Wirtz et al. [34]. A sample was considered positive when its optical density (OD) value was two-fold higher than the mean optical density of negative control.

#### Molecular identification of species of the *Anopheles gambiae* complex

A sample of 50 females belonging to the *An. gambiae* complex were randomly selected after each collection period for DNA extraction. DNA was extracted from the legs, abdomen or wings using the Livak protocol [35]. The SINE PCR [36] was performed to identify members of the *An. gambiae* complex.

#### Analysis

The human biting rate (HBR) was estimated as the number of mosquitoes collected per man per night. The sporozoite infection rate (or circumsporozoroite rate) was calculated by dividing the number of female anopheline found infected by the total number of mosquitoes screened. The daily entomological inoculation rate (EIR) was calculated by multiplying the human biting rate by the circum sporozoite rate. Comparisons of proportion were made using the Chi-square test. The level of significance of each test was set at P < 0.05.

## Results

### Composition of *Culicidae fauna* in the different study sites

**Fig. 2:**
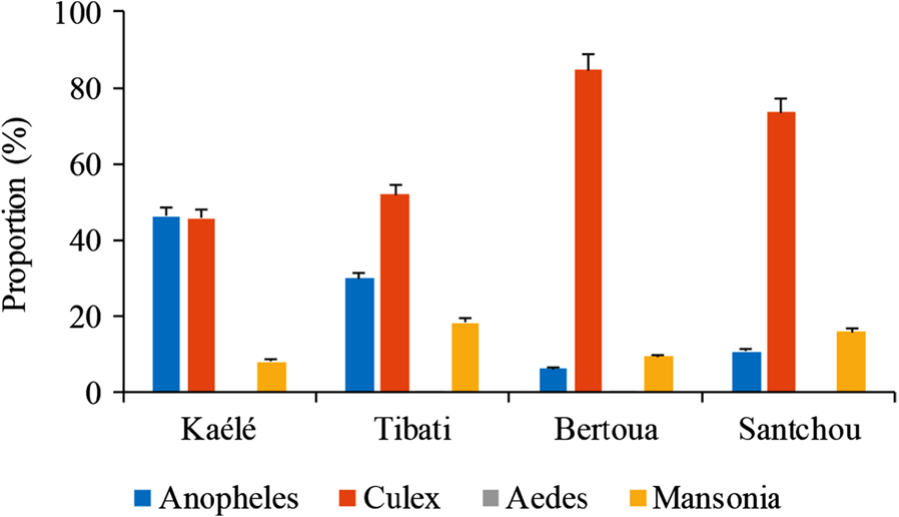
Distribution of mosquito genera in the different study sites

A total of 23,533 mosquitoes were collected during the study period (Fig. 2 and Additional file 1: Table S1). Four genera were recorded after morphological identification namely *Culex, Mansonia, Anopheles* and *Aedes.* The genus *Culex* was the most represented (59.83%; n = 14,079). Anopheline represented 28.31% (6663/23,533) of the total mosquitoes collected. Kaélé had the highest number of Anopheles specimens caught (46.17%; n = 4484) followed by Tibati (29.83%; n = 1449), Santchou (10.63%; n = 446) and Bertoua (5.95%; n = 284).

### Composition of the *Anophelinae* fauna in the different study sites

**Fig. 3:**
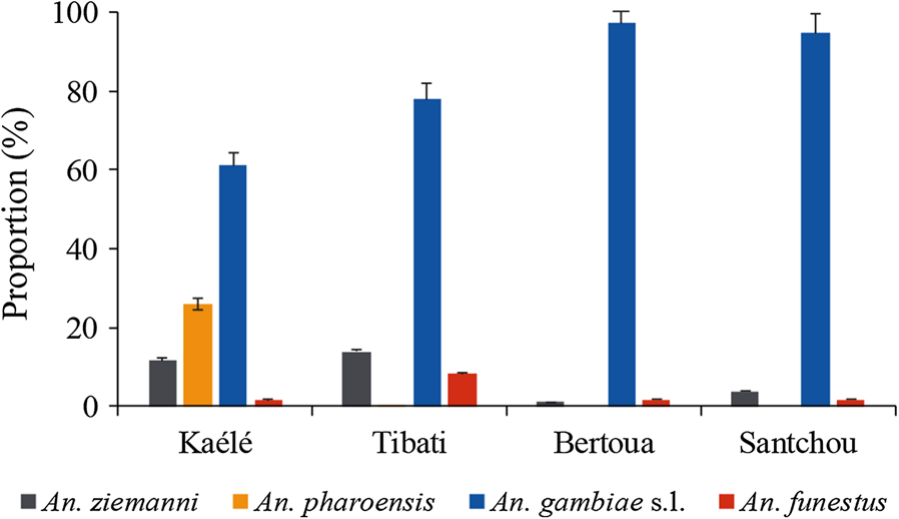
Distribution of *Anopheles* species in the different study sites

A total of 6666 *Anopheles* mosquitoes belonging to six anopheline species, including *An. gambiae.*, *Anopheles* *coluzzii*, *Anopheles arabiensis, Anopheles ziemmani, Anopheles* *pharoensis* and *Anopheles* *funestus* were collected (Table 2). *Anopheles gambiae s.l.* was the most abundant in all collection sites representing 68.54% (4567/6666) of the total anopheline fauna (Fig. 3 and Additional file 1: Table S2).

**Table 2:** Distribution of members of the *An. gambiae* complex in Kaélé, Tibati, Santchou and Bertoua

Species	Kaélé N (%)	Tibati N (%)	Sites	Santchou N (%)	Total N (%)
			Bertoua N (%)		
*An. gambiae*	126 (50.40)	54 (21.60)	96 (64)	108 (72)	384 (48)
*An. coluzzii*	90 (36)	175 (70)	54 (36)	13 (8.67)	332 (41.5)
*An.arabiensis*	30 (12)	13 (5.20)	0 0)	0 (0)	43 (5.38)
*An. gambiae* × *An. coluzzii*	4 (1.60)	8 (3.20)	0 (0)	29 (19.33)	41 (5.12)
Total	250 (100)	250 (100)	150 (100)	150 (100)	800 (100)

**Fig. 4:**
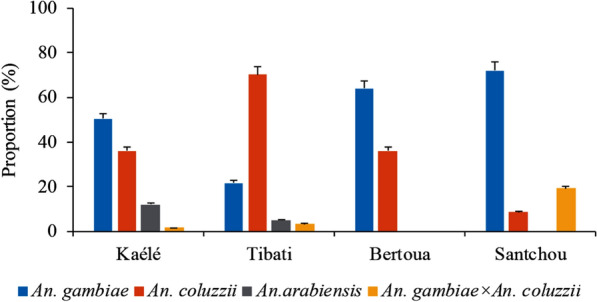
Distribution of members of the *An. gambiae* complex in the different study sites

A total of 800 out of the 4567 *An. gambiae s.l.* were further processed by PCR to identify sibling species of the *An. gambiae* complex in each site. *Anopheles gambiae*, *An. coluzzii* and *An. arabiensis* were recorded. *Anopheles gambiae* sensu stricto (*s.s*.) was the most frequent member of the group in Kaélé (50.40%; n = 126/250), Santchou (72%; n = 108/150) and Bertoua (64%; n = 96/150). *Anopheles coluzzii* was more frequent in Tibati (70%; n = 175/250 (Fig. 4 and Additional file 1: Table S3). *Anopheles arabiensis* was recorded in low frequencies exclusively in Kaélé (12%; n = 30/250) and Tibati (5.20%; n = 13/250).

### Indoors and outdoors biting Anopheline densities in different study sites

**Fig. 5:**
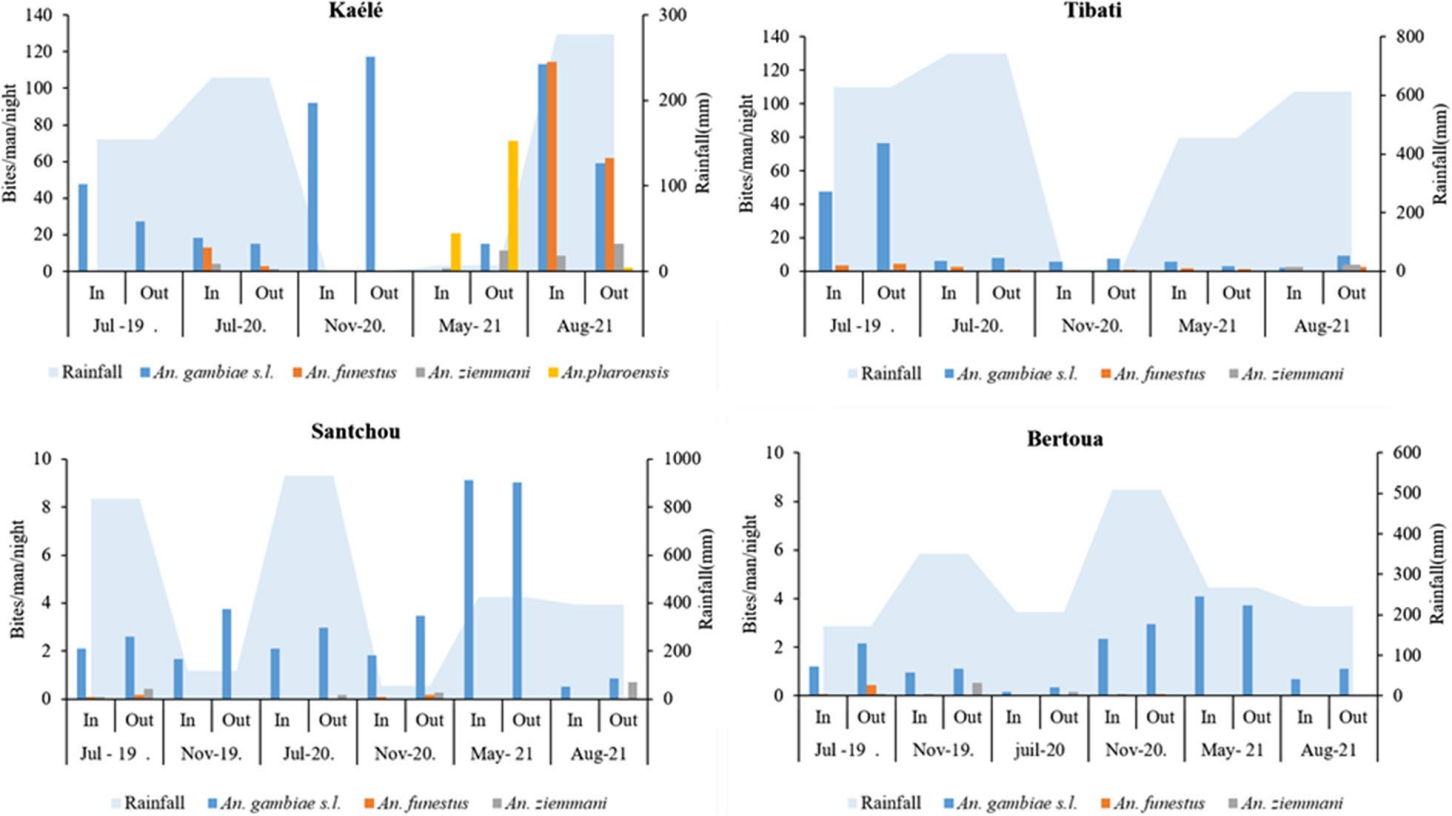
Distribution of anopheline species indoor and outdoor according to collection periods (In: indoor; Out: outdoor)

The proportion of female anopheline mosquitoes caught outdoor was 57.97% (n = 1449) in Tibati; 62.69% (n = 446) in Santchou, 60.35% (n = 285) in Bertoua and 48.48% (n = 4484) in Kaélé. These proportions were significantly higher compared to indoor catches in Tibati (χ^2^ = 35.27; Df = 1; P < 0.0001), Santchou (χ^2^ = 26.05; Df = 1; P = 0.001) and Bertoua (χ^2^ = 10.89; Df = 1; P < 0.0001). The density of anopheline was linked to rainfall and seasons. High densities occur during the rainy season with the predominance of *An. gambiae s.l.* in all sites (Fig. 5).

In Kaélé, *An. gambiae s.l.* was the predominant species during collection done in July 2019, November 2020 and August 2021 with biting rate reaching 122.75 b/m/n. In 2021, *An. gambiae* and *An. pharoensis* were recorded at similar densities and were the most predominant species (Fig. 5a). In Tibati, a peack of bite for *An. gambiae s.l.* (125 b/m/n) was recorded in July 2019 but a total change in biting densities was detected in August 2021 with *An.* *ziemmani* becoming the most abundant species (Fig. 5b). In Bertoua and Santchou, *An. gambiae s.l.* was always the most abundant species with biting densities varying from 0.1 to 8.74 b/m/n in Santchou and 0.1 to 3.91 b/m/n in Bertoua (Fig. 5c, d).

### Night biting cycle of *Anopheline* species

**Fig. 6:**
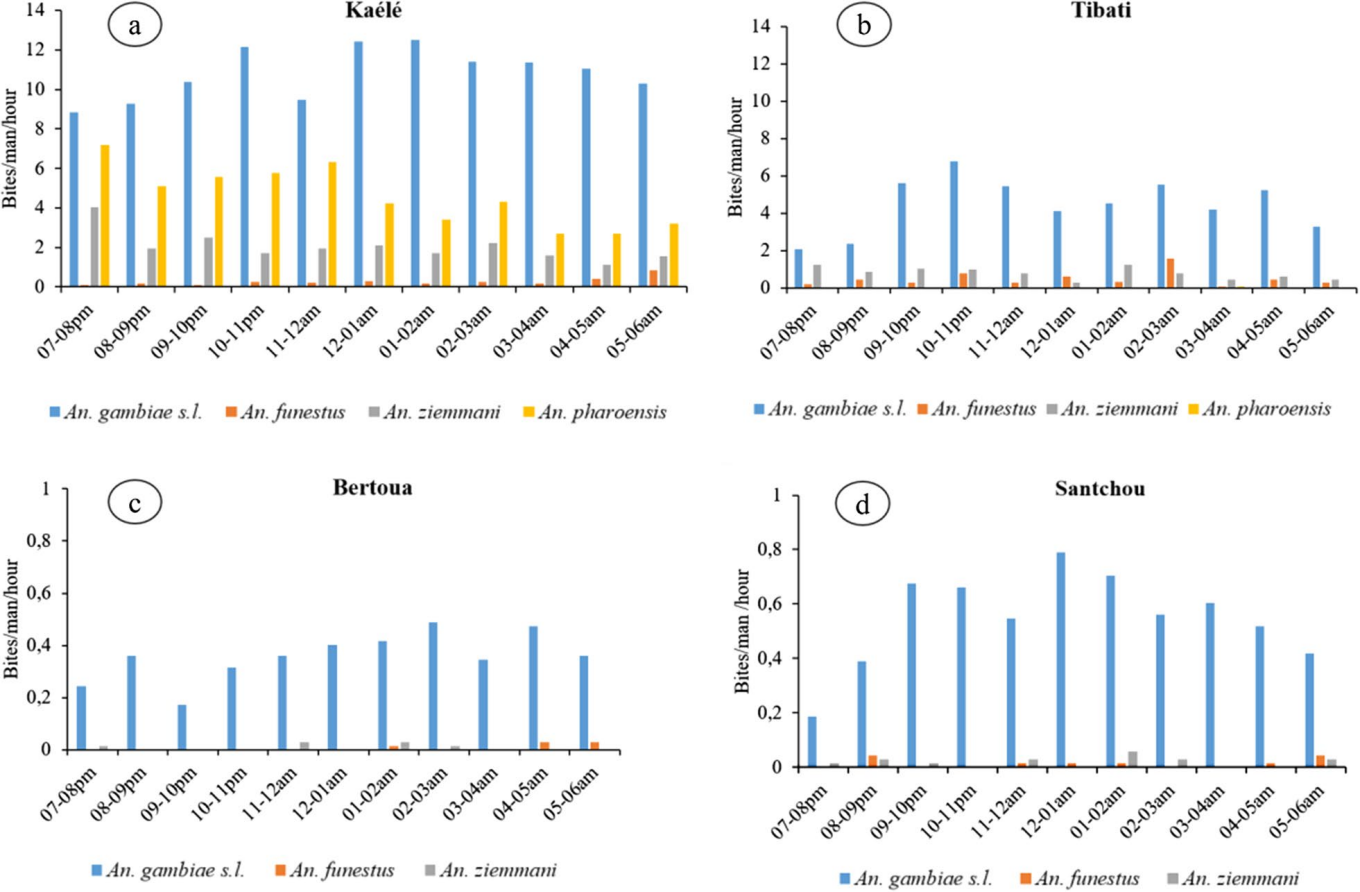
Biting cycles of *Anophelines* species in different study sites

The night biting cycle of anopheline species are presented in Fig. 6. In Santchou (Fig. 6d) and Bertoua (Fig. 6c) most of anopheline bites were recorded during the second half of the night (from 00:00 to 6:00 am) whereas in Tibati and Kaélé (Fig. 6a, b), most of the bites were recorded during the first half of the night (from 7:00 pm to 00:00 am).

### Plasmodium infection

A total of 4889 mosquitoes were screened to detect the presence of *Plasmodium* infections. After analysis, 36 Anopheline specimens were found infected representing a global infection rate of 0.74%. Both *An. gambiae s.l.* and *An. pharoensis* were detected infected in Kaélé with infection rates of 0.36% (6/1645) and 0.20% (2/1028) respectively. In Santchou, both *An. gambiae s.l.* and *An.* *ziemmani* were detected infected with sporozoite rate of 2% (6/309) and 16.70% (1/6) respectively*.* In Tibati and Bertoua, only *An.gambiae* *s.l*. was found infected, with sporozoite rate of 0.80% (10/1285) in Tibati and 4% (6/151) in Bertoua (Table 3).

**Table 3:** Sporozoite infection rates of Anopheles mosquitoes collected in Kaélé, Tibati, Santchou and Bertoua

Localities	Kaélé			Tibati			Santchou			Bertoua		
Species	Tested	Infected	% (95%CI)	Tested	Infected	%(95%CI)	Tested	Infected	% (95%CI)	Tested	Infected	% (95%CI)
*An. gambiae s.l*	1.645	6	0.36 (0.34–0.4)	1.285	10	0.80 (0.73–0.82)	309	6	2 (1.8–2.10)	151	6	4 (3.66–4.30)
*An. ziemmani*	74	0	0	15	0	0	6	1	16.70 (13.56–20.75)	3	0	0
*An. pharoensis*	1.028	2	0.20 (0.17–0.22)	2	0	0	0	0	0	0	0	0
*An. funestus*	68	0	0	110	0	0	7	0	0	5	0	0
*Total*	2.815	8	0.28 (0.26–0.30)	1.412	10	0.71 (0.66–0.75)	322	7	2.17 (2.01–2.34)	159	6	3.77 (3.46–4.09)

### Entomological inoculation rates

**Fig. 7:**
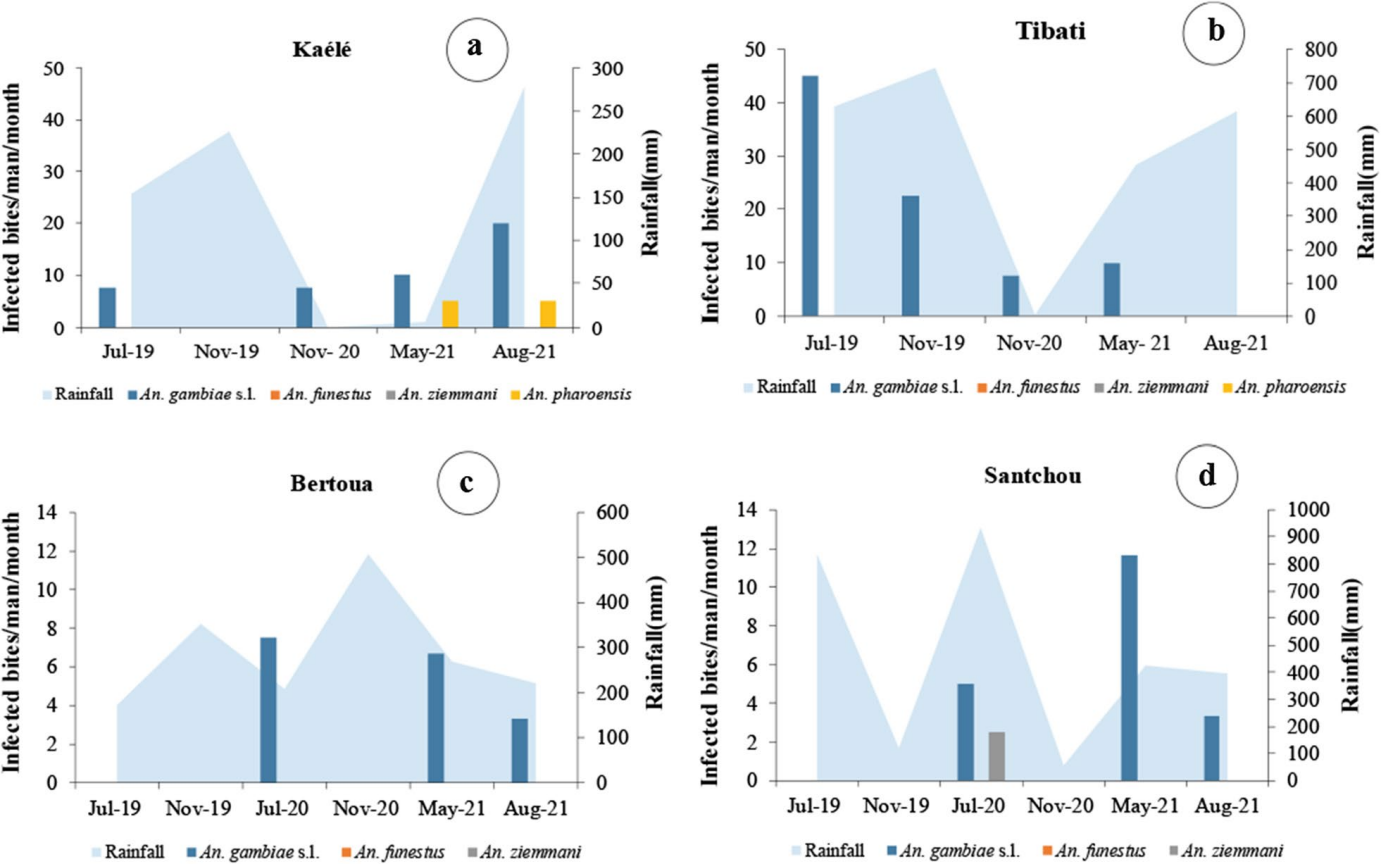
Monthly variation of the entomological inoculation rates in different study sites

The average daily entomological inoculation rate (EIR) was 0.26 ib/m/n in Kaélé and 0.21 ib/m/n in Tibati, 0.08 ib/m/n in Bertoua and 0.07 ib/m/n in Santchou (Table 4). Seasonal variation of the EIR was observed in all sites. *An. gambiae* was involved in malaria transmission in all sites across all the periods (Fig. 7).

**Table 4:** Daily entomological inoculation rate (EIR) in each study site

	Kaélé	Tibati	Bertoua	Santchou
Total collected	4484	1449	285	446
Biting rate (b/m/n)	93.41	30.18	2.17	2.17
Average sporozoite rate(%)	0.2	0.78	3.77	3.97%
Average daily EIR (ib/m/n)	0.26	0.21	0.08	0.07
Average yearly EIR (ib/m/y)	94.9	76.65	29.20	25.55

In Kaélé, *An. gambiae s.l.* was responsible for malaria transmission, except in November 2019, marking the beginning of the dry season. *Anopheles* *pharoensis* was found infected during the months of May and August 2021. Annual transmission was estimated at 95 infected bites/man/year (Fig. 7a). The highest transmission occurs in August 2021 during the rainy season.

In Tibati, transmission was mainly due to *An. gambiae s.l.* except August 2021. Annual transmission was estimated at 76.65 ib/m/y (Fig. 7b). The highest transmission was recorded in July 2019 during the pick of the rainy season.

In Santchou, malaria transmission was ensured by *An. gambiae s.l.* and *An. ziemmani* during the periods of July 2020 and May 2021. Annual transmission was estimated at 25.55 ib/m/y. The month of May 2021 in the mid of the rainy season was associated with high EIR (11.66 ib/m/month) maintain by *An. gambiae* (Fig. 7d).

In Bertoua, only *An. gambiae s.l.* was recorded as vector and transmission was registered in July 2020, May 2021 and August 2021. Annual transmission rate in the locality was estimated at 29.20 ib/m/y. The month of July 2020 during the short dry season was associated with the highest EIR (7.50 ib/m/y) (Fig. 7c).

## Discussion

The main objective of the study was to assess the dynamic of malaria transmission in sentinel sites within four ecoepidemiological settings in Cameroon including the Sahelian (Kaélé), Soudano-sahelian (Tibati), highland (Santchou) and forest zone (Bertoua). Despite high possession and usage of ITNs in studied sites [6], continuous transmission of malaria was recorded. Transmission estimates recorded during this study were similar to data recorded in other parts of the country [8, 13–15]. Yet very high transmission intensity was found to occur in both Kaélé and Tibati situated in Sahelian and Soudano-sahelian zones, respectively, compared with Santchou and Bertoua situated in the highland and forest area, respectively. This may result from the high biting anopheline densities in those areas particularly during the rainy season. The two sites are situated close to dams which might contribute in the maintenance of vector populations all year long. The EIR in Santchou was low, which is in line with previous findings supporting moderate malaria endemicity in the highland region [24, 37]. Malaria transmission intensity in Bertoua was also relatively moderate. Bertoua is situated in the East region of Cameroon considered as one of the most affected by malaria in the country with high morbidity and high mortality rates [38]. The moderate transmission pattern in the city of Bertoua may owe to the development of infrastructures such as drains and roads during past years.

Six Anopheline species were identified in this study, namely *An. gambiae s.s., An. coluzzii, An. arabiensis, An. ziemmani, An. pharoensis,* and *An. funestus*. The presence of different species was consistent with the diversity of aquatic habitats for mosquitoes in the studied sites including permanent water sources, puddles, dams, rivers. Species diversity was poor compared to previous reports [15] and probably reflects the effects of climatic or environmental changes on species distribution. Studies conducted so far in Cameroon point at a temperature increase of + 0.4 °C compared with the 1961–1990 period, and a rainfall reduction ranging from − 10 to − 20% [39]. *Anopheles* *gambiae s.l.* was the most abundant species and the major malaria vector in all sites. Previous studies indicated the high adaptation capacity of members of the complex to environmental conditions [32, 40–42]. Three members of the *An.* *gambiae* complex were detected, including *An. gambiae*, *An. coluzzii* and *An. arabiensis*. Both *An. gambiae* and *An. coluzzii* were found in sympatry in the four sites, whereas *An.* *arabiensis* was only detected in Kaélé and Tibati, with a low frequency. The distribution of members of the *An.* *gambiae* complex was consistent with previous studies mapping the distribution of these species in the country [40].

Other anopheline species, including *An.* *funestus*, *An.* *pharoensis* and *An. ziemmani*; were collected alongside members of the *An. gambiae* complex. Despite the high vectorial capacity of *An. funestus* this species was not found infected in any of the sites. However, this species has usually been found to display a high vectorial capacity compared to *An. gambiae* in different epidemiological settings [15, 19]. *Anopheles pharoensis* and *An. ziemmani* were found infected both in Kaélé and Santchou respectively. Considered as secondary vectors, these species were actively involved in malaria transmission. These findings are consistent with previous studies in Cameroon where up to 17 species were reported involved in malaria transmission in various places [15, 43, 44]. *Anopheles pharoensis* was collected in very high densities in Kaélé. The species is considered to breed in shallow, sunlight, freshwater pools with high organic content; the presence of rice paddies in Kaélé and a lake reservoir with a high vegetation on it edges could possibly constitute preferential breeding habitats for the species [45, 46]. The distribution of anopheline species in Kaélé could be highly dynamic since previous studies indicated high densities of *An. funestus* in the area [47], whereas no *An. funestus* was collected during the present study. It is possible that the reduction of land surface used for rice cultivation, extension of human settlements following the severe drought which affected the region since 2017 and population displacement following Boko-haram insurgency alongside new agricultural practices are shaping species distribution and malaria transmission patterns within the area [48–50]. The situation deserves further investigations. *Anopheles ziemmani* was recorded in high density in both Tibati and Kaélé this species was mostly collected outdoors. *Anopheles ziemmani* has so far received less attention despite its implication in malaria transmission in Cameroon [15]. The species belongs to a group composed of *Anopheles coustani, Anopheles namibiensis, Anopheles* *obscurus* and *Anopheles implexus* with some being highly zoophilic and not involved in malaria transmission at all [51].

Anopheline species were frequently caught biting outdoors in almost all sites except in Kaélé. This behaviour might result from the high ownership and usage of insecticide treated bed nets by the population [6]. This is in line with previous studies in Cameroon, indicating high transmission of malaria both indoor and outdoor [11, 27]. This trend was also described in many other African countries [14, 52, 53]. In Kaélé, anopheline were found to bite mostly indoor. Due to the high nuisance in the area the population usually stay indoor during the night and this behaviour might have influenced mosquito biting behaviour. It is possible that the high frequency of resistant mosquito in the area could also explain why mosquitoes in the area are not affected by the excito-repellency effects of insecticide treated bed nets.

Mosquitoes were found to bites all night long with picks in Kaélé and Tibati recorded during the first half of the night and during the second half of the night in Bertoua and Santchou. *Anopheles pharoensis* and *An.* *ziemmani* were found to bite predominantly during the first part of the night, whereas *An. gambiae s.l.* was found to bite both in the first and second part of the night. Studies conducted so far in Cameroon indicated that mosquitoes could bite up to 8 am [54] although for the moment there is still no data on the epidemiological consequences of this behaviour. This calls for further actions to improve malaria vector control in the country.

## Conclusion

This study of the dynamics of malaria transmission in different eco-epidemiological settings shows a heterogeneous pattern of malaria transmission. Malaria transmission was found to be high in all eco-epidemiological settings with different factors contributing to transmission. The high presence of vectors biting outdoors calls for urgent actions in order to improve the fight against malaria in Cameroon. Additional tools to control outdoor biting mosquitoes should be implemented (larviciding, environmental management). Integrated vector management could constitute an efficient way to fight against continuous malaria in the country and achieve elimination goals.

## Supplementary Information

**Additional file 1: Table S1.** Distribution of mosquito genera in the different study sites. **Table S2.** Distribution of Anopheles species in the different study sites. **Table S3.** Distribution of members of the *An. gambiae* complex in different study sites.

## Data Availability

The datasets supporting the findings of this paper are included in this paper.
