# Supplementary material for: Malaria transmission pattern across the Sahelian, humid savanna, highland and forest eco-epidemiological settings in Cameroon
Source: Malar J. 2023 Apr 7;22:116. doi: 10.1186/s12936-023-04544-z (PMC10080520; doi:10.1186/s12936-023-04544-z)
Supplement: Supplementary file 1 — Additional file 1: Table S1. Distribution of mosquito genera in the different study sites. Table S2. Distribution of Anopheles species in the different study sites. Table S3. Distribution of members of the An. gambiae complex in different study sites. [file 12936_2023_4544_MOESM1_ESM.docx]

**Table S1:** Distribution of mosquito genera in the different study sites.

|  | Kaélé | Tibati | Bertoua | Santchou | Total |
| --- | --- | --- | --- | --- | --- |
| Genera | N (%) | N (%) | N (%) | N (%) | N(%) |
| *Anopheles* | 4,484 (46.17) | 1,451 (29.87) | 285 (5.97) | 446 (10.63) | 6,666 (28. 32) |
| *Culex* | 4,440 (45.72) | 2,514 (51.75) | 4,042 (84.73) | 3,083 (73.47) | 1,4079 (59.82) |
| *Aedes* | 0 (0) | 6 (0.12) | 1 (0.02) | 0 (0) | 7 (0.03) |
| *Mansonia* | 788 (8.11) | 887 (18.26) | 442 (9.27) | 667 (15.90) | 2,784 (11.83) |
| Total | 9,712 (100) | 4,858 (100) | 4,770 (100) | 4,196 (100) | 23,536 (100) |

**Table S2:** Distribution of Anopheles species in the different study sites**.**

|  | Kaélé | Tibati | Bertoua | Santchou | Total |
| --- | --- | --- | --- | --- | --- |
| Species | N (%) | N (%) | N (%) | N (%) | N (%) |
| *An. ziemmani* | 516 (11.50) | 199 (13.73) | 3 (1.05) | 17 (3.82) | 735 (11.03) |
| *An. pharoensis* | 1,160 (25.87) | 2 (0.14) | 0 (0) | 0 (0) | 1,162 (17.43) |
| *An. gambiae* s.l. | 2,740 (61.11) | 1,129 (77.91) | 277 (97.19) | 421(94.61) | 4,567 (68.54) |
| *An. funestus* | 68 (1.52) | 119 (8.21) | 5 (1.75) | 7 (1.57) | 199 (2.98) |
| Total | 4,484 (100) | 1,449 (100) | 285 (100) | 445 (100) | 6,663 (100) |

**Table S3:** Distribution of members of the *An. gambiae* complex in different study sites.

|  |  |  | Sites |  |  |
| --- | --- | --- | --- | --- | --- |
| Species | Kaélé  N (%) | Tibati  N (%) | Bertoua  N (%) | Santchou  N (%) | Total  N (%) |
| *An. gambiae* | 126 (50.40) | 54 (21.60) | 96 (64) | 108 (72) | 384 (48) |
| *An. coluzzii* | 90 (36) | 175(70) | 54 (36) | 13 (8.67) | 332 (41.5) |
| *An.arabiensis* | 30 (12) | 13 (5.20) | 0 (0) | 0 (0) | 43 (5.38) |
| *An. gambiae×An. coluzzii* | 4 (1.60) | 8 (3.20) | 0 (0) | 29 (19.33) | 41(5.12) |
| Total | 250 (100) | 250 (100) | 150 (100) | 150 (100) | 800 (100) |
